# Controlling thermal emission with refractory epsilon-near-zero metamaterials via topological transitions

**DOI:** 10.1038/ncomms11809

**Published:** 2016-06-06

**Authors:** P. N. Dyachenko, S. Molesky, A. Yu Petrov, M. Störmer, T. Krekeler, S. Lang, M. Ritter, Z. Jacob, M. Eich

**Affiliations:** 1 Institute of Optical and Electronic Materials, Hamburg University of Technology, Eissendorfer Strasse 38, Hamburg 21073, Germany; 2 University of Alberta, Department of Electrical and Computer Engineering, 9107—116 Street, Edmonton, Canada T6G 2V4; 3 ITMO University, 49 Kronverskii Avenue, Saint Petersburg 197101, Russia; 4 Institute of Materials Research, Helmholtz-Zentrum Geesthacht Centre for Materials and Coastal Research, Max-Planck-Straße 1, Geesthacht 21502, Germany; 5 Electron Microscopy Unit, Hamburg University of Technology, Eissendorfer Strasse 42, Hamburg 21073, Germany; 6 Birck Nanotechnology Center, School of Electrical and Computer Engineering, Purdue University, West Lafayette, Indiana 47906, USA

## Abstract

Control of thermal radiation at high temperatures is vital for waste heat recovery and for high-efficiency thermophotovoltaic (TPV) conversion. Previously, structural resonances utilizing gratings, thin film resonances, metasurfaces and photonic crystals were used to spectrally control thermal emission, often requiring lithographic structuring of the surface and causing significant angle dependence. In contrast, here, we demonstrate a refractory W-HfO_2_ metamaterial, which controls thermal emission through an engineered dielectric response function. The epsilon-near-zero frequency of a metamaterial and the connected optical topological transition (OTT) are adjusted to selectively enhance and suppress the thermal emission in the near-infrared spectrum, crucial for improved TPV efficiency. The near-omnidirectional and spectrally selective emitter is obtained as the emission changes due to material properties and not due to resonances or interference effects, marking a paradigm shift in thermal engineering approaches. We experimentally demonstrate the OTT in a thermally stable metamaterial at high temperatures of 1,000 °C.

## Abstract (web summary)

The ability to control thermal radiation at high temperatures is of interest for thermal photovoltaics. Here, Dyachenko *et al*. engineer the epsilon-near-zero frequency of a metamaterial and connected optical topological transition to selectively enhance and suppress the thermal emission in the near-infrared spectrum.

## Graphical abstract

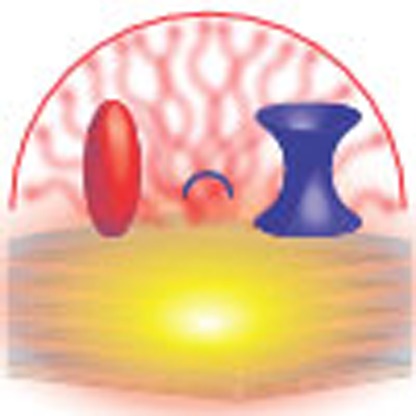

The photonic isofrequency surface relates the momentum and energy of optical modes inside a medium and can be engineered using metamaterials^1^. As opposed to regular spherical and ellipsoidal shapes seen in natural dielectrics, we can engineer exotic isofrequency surfaces in metamaterials. Examples include point-like vanishing surfaces as in epsilon-near-zero (ENZ) media^2^ and open surfaces as in hyperbolic media^3^. These surfaces support unique electromagnetic modes that can be used in sub-diffraction imaging^4^ and waveguiding^5^, spontaneous emission engineering^6^ and nanoscale resonators^7^. An important experimental problem is the thermal excitation of metamaterial modes lying on this unique photonic isofrequency surface for practical thermal applications.

Our aim in this paper is to demonstrate a high-temperature metamaterial with wavelength selective thermal emission arising from the ENZ behaviour associated with the optical topological transition (OTT)^1^ in the photonic isofrequency surface. This marks an important departure from well-established routes of two-dimensional and three-dimensional photonic crystals^8^,^9^,^10^,^11^,^12^,^13^,^14^,^15^,^16^,^17^, thin film resonances^18^,^19^,^20^,^21^, gratings and metasurfaces^22^,^23^,^24^,^25^,^26^,^27^, since we control an intrinsic material property and bulk thermal energy density to selectively excite and suppress bulk metamaterial modes that contribute to far-field thermal emission. Furthermore, we do not utilize resonances or bandgap effects and achieve the effect by tuning the ENZ frequency (plasma frequency) of a metamaterial. We show that the experimentally observed far-field thermal radiation spectrum is a unique signature of the change in the reflectivity of the metamaterial that occurs due to the topological transition in the isofrequency surface. The metamaterial is designed based on a subwavelength super-lattice structure with refractory materials tungsten and hafnium oxide in stark contrast to previous ENZ and hyperbolic media which have utilized noble metals with low-temperature stability^28^, phonon-polaritonic materials^29^, graphene^30^,^31^ and highly doped semiconductors^32^. We conclusively demonstrate the high-temperature stability of the optical absorption and thermal emission at temperatures of 1,000 °C. The OTT is carefully designed to lie in the near-infrared window (1–3 μm wavelength) paving the way for refractory metamaterials compatible with low-bandgap photovoltaic (PV) cells^33^,^34^,^35^,^36^ (0.3–0.6 eV). We strongly emphasize that the unique selective thermal emission spectrum at the OTT is desirable for high-efficiency thermophotovoltaic (TPV) system proposals^37^,^38^,^39^,^40^. In exchange for improved conversion efficiency and application flexibility the implementation of the TPV concept places stringent requirements on the emissivity characteristics and thermal stability of the selective thermal emitter, Fig. 1a. Thermodynamic conservation arguments require the radiative thermal emissivity of any object to be less than that of a blackbody at the same temperature. Consequently, to achieve sufficient output radiative power density in the range of contemporary low-bandgap photovoltaic receivers^33^ with bandgaps of typically 0.55 eV operational temperatures surpassing 1,000 °C are required. The emitter should also possess the highly selective emissivity characteristics required to suppress the emission of long-wavelength photons and, at the same time, should provide near unity emissivity at energies above the bandgap of the PV cell, Fig. 1a. Short wavelength selectivity of the emitter is not required since the blackbody limit at 1,000 °C represents a natural boundary due to the Bose–Einstein occupation quickly decreasing for shorter wavelengths.

Our design has low angular dependence and is tunable within the entire near-infrared spectrum. We also reveal the mechanisms leading to thermal degradation of the optical performance through spectral analysis and energy-dispersive X-ray spectroscopy (EDS) techniques. Our measurements of the optical absorption and thermal emission at high temperature (1,000 °C) serve as a direct validity of Kirchhoff's laws^41^ for bulk effective medium parameters and pave the way for refractory thermal metamaterials. The OTT is obtained in the vicinity of natural material excitations such as plasmons and optical phonons. However, utilizing natural material resonances for thermal emission engineering poses severe challenges as outlined in Fig. 1b. Conventional plasmonic materials suffer from low melting points and from too high plasma frequencies^42^,^43^, while phonon-polaritonic materials^29^ have resonances in the mid-infrared range that cannot be moved easily to higher energies in the 2–4 μm wavelength region crucial for high-temperature nanophotonic applications such as TPV. Thus metamaterials are required to shift OTT to the required spectral range.

## Results

### Design of metamaterial

Our aim is to engineer an OTT in the near-infrared spectral window with refractory building blocks to control thermal emission. The OTT is a change in the isofrequency surface of a metamaterial which changes from a closed ellipsoid to an open hyperboloid and affects fundamental properties such as the photonic density of states and bulk thermal energy density^44^,^45^. In our case the hyperbolic isofrequency surface does not provide modes that can be coupled out of the metamaterial to the far field and thus the emission at longer wavelengths is naturally suppressed. The OTT is a material approach to achieve either enabling or suppression of the thermal emission since the two isofrequency surfaces lead to very different optical reflectivities. According to Kirchhoff's law a body's radiation absorptivity and thermal emissivity are identical. Thus, we consider calculated and measured absorptivity and emissivity as equal throughout the text. As a consequence the sharp change in reflectivity which affects the optical absorption spectrum also dictates the thermal emission spectrum of the metamaterial.

The simplest realization of a metamaterial with a controllable topological transition consists of alternating subwavelength layers of metal and dielectric, as displayed schematically in Fig. 2c. Figure 2d is the scanning electron microscopic (SEM) image of the fabricated structure. The effective medium response of this basic layered structure is given by the weighted arithmetic average along the material planes, ɛ_||_=(*d*_M_*ɛ*_M_+*d*_D_*ɛ*_D_)/(*d*_M_+*d*_D_) and the weighted harmonic average along the optical axis, 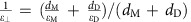. Note that the effective medium parameters, controlling the topological transition, are determined simply by the permittivities of the individual layers, *ɛ*, and their thicknesses, *d*_M_ and *d*_D_, within the unit cell. The ‘D' and ‘M' subscripts used here denote dielectric and metallic materials. The metamaterial consists of refractory metal tungsten (20 nm layers) and a transparent dielectric hafnium dioxide (100 nm layers) on a 100-nm tungsten substrate as shown in Fig. 2c,d. Our refractory material choices with their high-temperature stabilities are unique for multilayer metamaterials. Figure 2b shows the excellent agreement between dielectric parameters extracted using spectroscopic ellipsometry and an effective medium anisotropic model. It should be noted that the OTT occurs at the ENZ wavelength (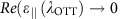) when the real part of the perpendicular dielectric constant changes sign from positive (dielectric) to negative (metal). Furthermore, we emphasize the striking feature that the OTT survives even in the presence of high losses present in tungsten.

Figure 2a shows the predicted emissivity of the tungsten–hafnium dioxide multilayer calculated using transfer matrix theory. We emphasize that the topological transition is captured completely by an effective medium theory (EMT). EMT simulations take into consideration the presence of the substrate and the topmost capping layer to account for the finite number of layers forming the metamaterial. The calculated absorptivity of the presented metamaterial with four periods shows <1% difference compared with the absorptivity of the same metamaterial with 20 periods. Thus, the transmissivity *T* is negligible and the designed and fabricated number of layers represents a satisfactory approach to determine the absorptivity and emissivity of an infinite metamaterial.

Figure 3 analyses the angular dependence of the absorptivity of the metamaterial. The angular-dependent emissivity of the structure is the power per area, thermally radiated at a specific angle and wavelength interval divided by the same quantity of an ideal blackbody. As seen in Fig. 3, the calculated large absorptivity thus emissivity at a given wavelength between 1.0 and 1.5 μm is largely independent of the angle of incidence or angle of observation up to angles of ∼70°. For larger angles Fresnel reflectivity is increasing and the absorptivity and emissivity drop down to zero. As photons at such large angles will not be emitted no effective heat loss of the source takes place and thus the efficiency of an envisaged TPV-system will not be altered by this fact.

Along with spectral selectivity, this is an important characteristic desired for practical TPV applications. In a TPV-system, an omnidirectional emitter is clearly advantageous as it allows extracting and transmitting a much larger radiative power. The dark regions correspond to low thermal emissivity in a broad angular range beyond the OTT as predicted by our effective medium approach whereas the white regions depict high thermal emissivity.

We now discuss the coupling of the thermally excited metamaterial modes with vacuum, which determines the thermal radiation spectrum. The OTT between the closed ellipsoidal and open hyperboloidal isofrequency surface occurs at the ENZ frequency. At wavelengths below the topological transition, the wavevectors of thermally excited modes lie on the surface of the ellipsoid and have tangential components matched to that of vacuum modes. Thus the thermally excited modes can efficiently couple to vacuum modes maximizing the power density of thermal emission (Fig. 2a). Above the topological transition wavelength, the isofrequency surface of the refractory metamaterial forms a type II hyperboloid^6^, supporting only extraordinary modes with very large wavevectors which cannot be matched to vacuum modes. Thus there is no coupling between the hyperbolic modes and the vacuum modes leading to a strong suppression of radiative infrared thermal emission. This suppression of thermal emission at low energies beyond the ENZ frequency is critical to TPV-efficiency enhancement since these are sub-bandgap photons not absorbed by the cell. We emphasize that the tungsten/hafnium oxide layers are deeply subwavelength and the super-lattice functions in the effective medium limit for temperatures of 1,000 °C since the relevant blackbody spectrum is in the near-infrared range.

### Experimental verification

**Figure 1:**
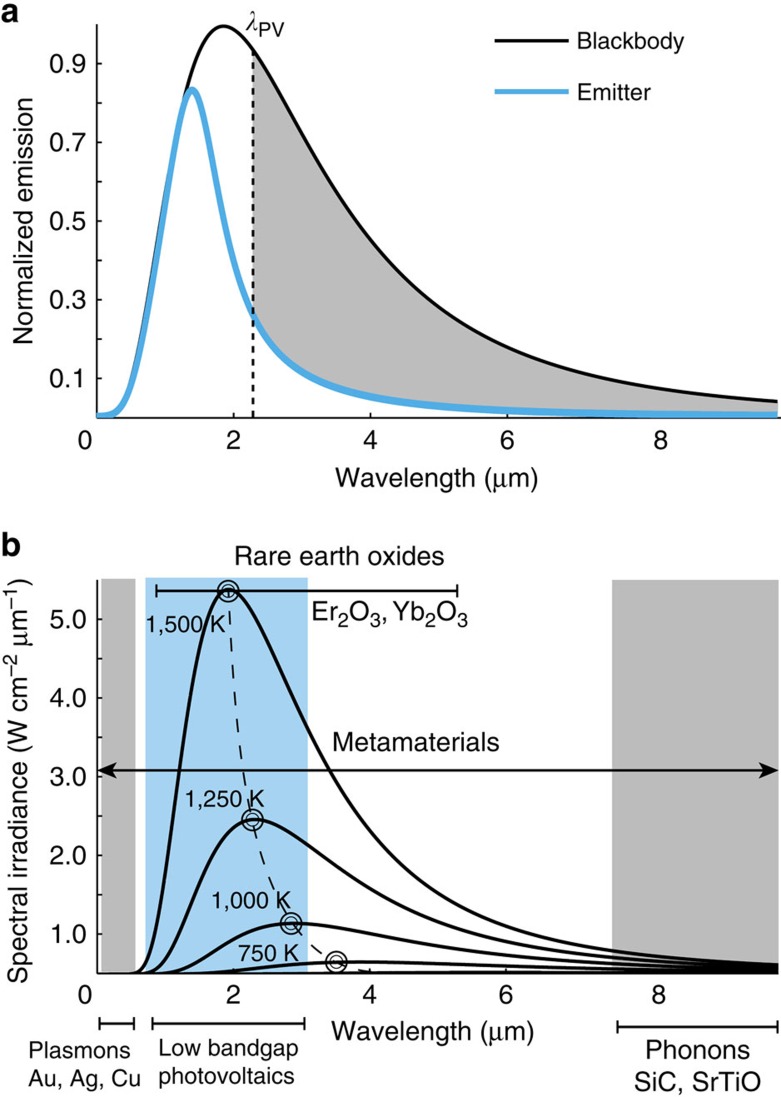
Selective thermal emitters for thermophotovoltaics. (**a**) The blackbody emission at 1,000 °C (1,273 K) normalized to its maximum and the emission of a selective TPV emitter at the same temperature normalized to the maximum of the blackbody emitter are presented. The selective emitter suppresses thermal emission throughout the infrared and simultaneously provides near blackbody emission at energies above the bandgap of the PV cell. The band-edge of a PV cell with 0.55 eV is presented by the dashed line indicating *λ*_PV_. (**b**) Overlap of the spectral irradiance of a blackbody half-space with natural optical resonances. The blackbody spectrum for increasing temperatures between 750 and 1,500 K shows the peak lying in the near-infrared region for high temperatures (1,500 K) which is the spectral range for contemporary low-bandgap photovoltaics (blue shaded area). Note, these temperatures are necessary for high-efficiency energy conversion but are beyond the reach of conventional plasmonic building blocks for metamaterials because of their low melting point. On the other hand, thermal engineering approaches based on optical phonons are restricted to the mid-infrared spectrum and are difficult to move to the near-infrared range. Metamaterial principles extend the spectral range of bulk optical material resonances throughout the infrared.

**Figure 2:**
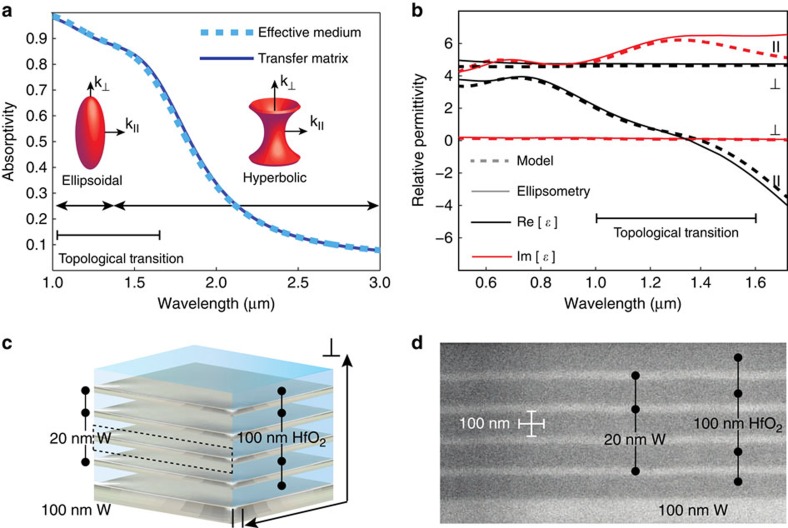
Concept and implementation of the tungsten/hafnium dioxide refractory metamaterial. (**a**) Calculated absorptivity characteristic of the metamaterial at normal incidence. At vacuum wavelengths below the topological transition the medium supports radiative modes resulting in high absorptivity. Beyond the transition the metamaterial allows only modes with large tangential components of the wavevector which cannot couple to optical modes propagating in vacuum. This leads to a strong suppression of the structure's absorptivity and thus of its emissivity. Because of the small unit cell size excellent agreement is seen between the effective medium and rigorous transfer matrix theory. (**b**) Comparison of the theoretically designed, based on permittivity data provided by Roberts^52^, and ellipsometrically extracted relative permittivity parameters for the metamaterial structure (see methods). In all simulations the hafnium dioxide layers are assumed to be lossless and dispersionless with a relative permittivity of *ɛ*=3.88. (**c**) Schematic image of the refractory metamaterial design. The dashed box shows the metamaterial unit cell. (**d**) SEM image of the fabricated refractory metamaterial. By choosing the thicknesses of the nano-structured refractory metal and oxidic dielectric layers, topological transitions can be tuned throughout the infrared.

**Figure 3:**
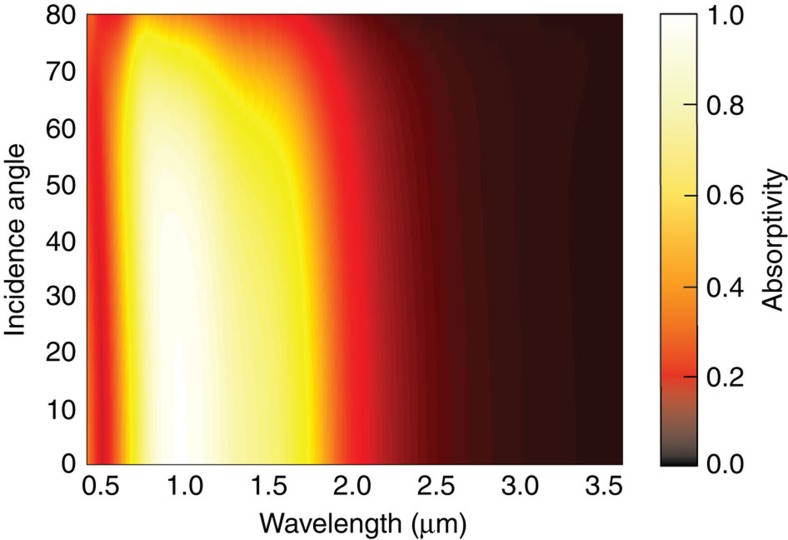
Absorptivity of the metamaterial versus incidence angle. Simulated absorptivity of the tungsten/hafnium dioxide metamaterial described in Fig. 2 as a function of wavelength of incident light and as a function of the angle of incidence, taking into account both s- and p- polarizations. The simulation is performed using the transfer matrix formalism assuming optical properties of tungsten from Roberts^52^, and that hafnium dioxide can be treated as a dispersionless dielectric with a relative dielectric constant of 3.88. The figure shows that the designed topological transition produces largely angularly independent thermal absorptivity up to the blackbody limit (white region in the colour plot) over a narrow spectral bandwidth suited to use with low-bandgap photovoltaic cells^33^,^34^,^35^,^36^. According to Kirchhoff's law and since the transmission through the metamaterial stack is negligible, the expected emissivity is considered identical to the calculated absorptivity. Because of the low coupling of modes between vacuum and hyperbolic regime of the metamaterial thermal radiation in the mid-infrared is strongly suppressed. Note that our approach is fundamentally different from structural resonances in metasurfaces and bandgap effects in photonic crystals.

**Figure 4:**
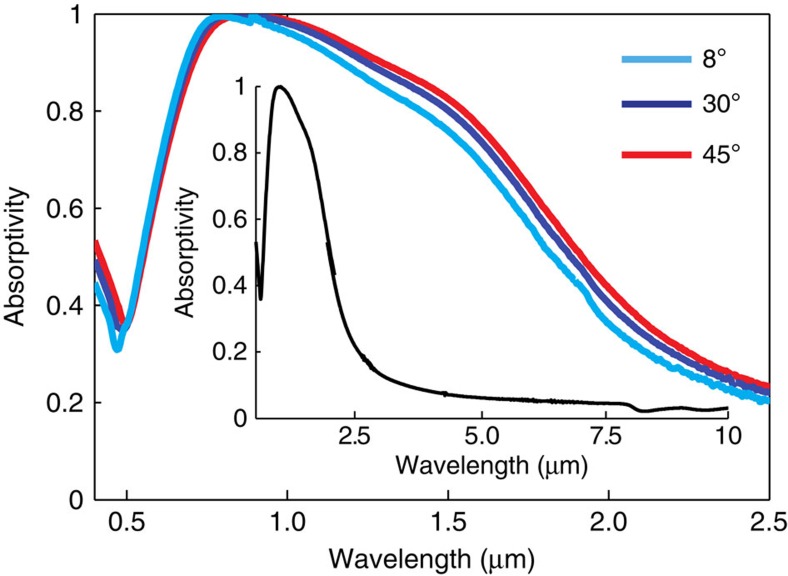
Measured absorptivity versus incidence angle. The absorptivity spectrum is obtained as *A(λ)=1–R(λ)–T(λ)*, where *A(λ)* is the spectral absorptivity, *R(λ)* the reflectivity of the metamaterial, and *T(λ)* is the transmissivity, which is negligible for the metamaterial with four periods. The measured absorptivity is in complete agreement with the theoretically predicted absorptivity presented in Fig. 3. Only reflectivity measurements are needed as we use a 100 nm thick tungsten substrate underneath the metamaterial which is intransparent over the measured wavelength range. The inset shows measured absorptivity of the tungsten/hafnium dioxide metamaterial at a 13^o^ angle of incidence from 0.5 to 10 μm. The observed long-wavelength absorptivity characteristics are in complete agreement with the effective medium calculation.

We first experimentally characterize the absorption spectrum of the ENZ metamaterial at room temperature for unpolarized light with incident angles between 0 and 45° (Fig. 4). The figure shows that the structure possesses both near unity angularly independent absorptivity at wavelengths around 800 nm up to the limit of the blackbody emission (Fig. 1) along with a strong suppression of absorptivity throughout the mid-infrared. Comparing with Figs 2a,b and 3, these observed characteristics are in complete agreement with the topological transition of the metamaterial design and the effective medium interpretation of the emitter structure. It should be noted that these characteristics are obtained by a route fundamentally different from tungsten photonic crystals^10^ which employ resonances to provide selective thermal emission.

**Figure 5:**
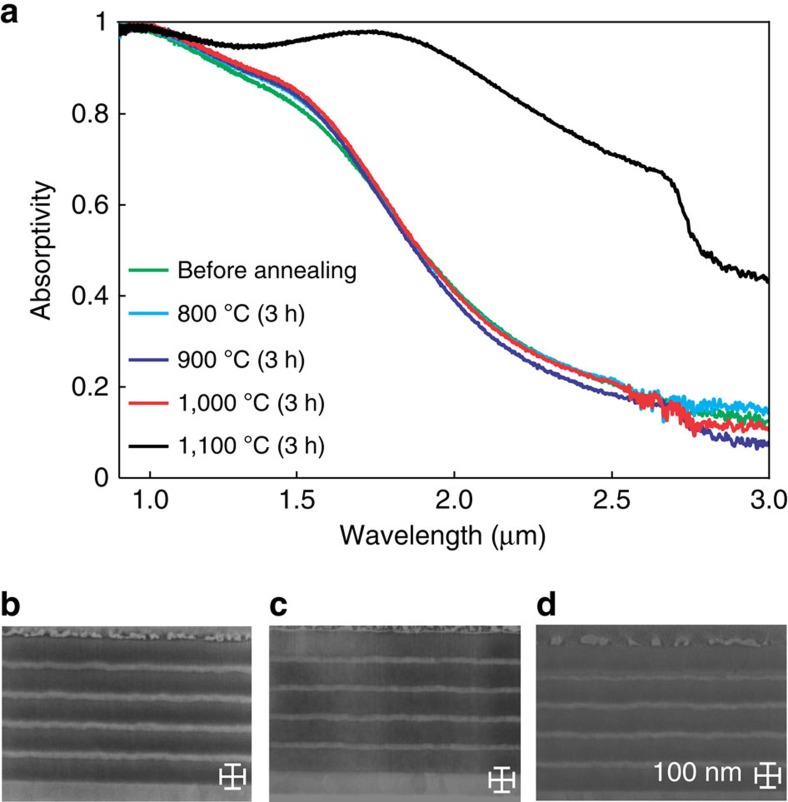
Thermal stability. (**a**) Measured absorptivity of the tungsten/hafnium dioxide metamaterial after annealing for periods of three hours each. Temperatures are displayed in the legend. (**b**–**d**) Cross-sectional SEM images of the metamaterial before annealing, after annealing at 1,000 °C for 3 h, and after annealing at 1,100 °C for 3 h respectively. Although the infrared absorptivity of the metamaterial is greatly altered after the 1,100 °C annealing step, no structural degradation is observed.

An important question is whether the topological transition in the refractory metamaterial is thermally stable after being subject to high temperatures. For this, we performed a series of increasingly harsh high-temperature annealing steps, each lasting multiple hours. Figure 5a compares the absorptivity spectra of the refractory metamaterial at room temperature before and after heat cycling to temperatures between 800 and 1,100 °C in vacuum. As shown, the optical characteristics of the metamaterial were practically unaffected by the high-temperature treatments, up to 3 h at 1,000 °C, demonstrating the heat cycle stability of the designed topological transition.

We pushed the annealing temperatures beyond 1,000 °C to explore the fundamental causes of degradation of optical properties at high temperatures. Significant deviations in the absorption spectrum were observed after annealing the emitter at 1,100 °C for a period of 3 h. To understand the irreversible degradation mechanism of the multilayer structure, SEM images of the sample were recorded before annealing (Fig. 5b), after annealing at 1,000 °C (Fig. 5c) and after annealing at 1,100 °C (Fig. 5d). These images show no structural degradation; however, chemical alterations, as shown below, indicating that higher temperature stability can be achieved with other material choices.

**Figure 6:**
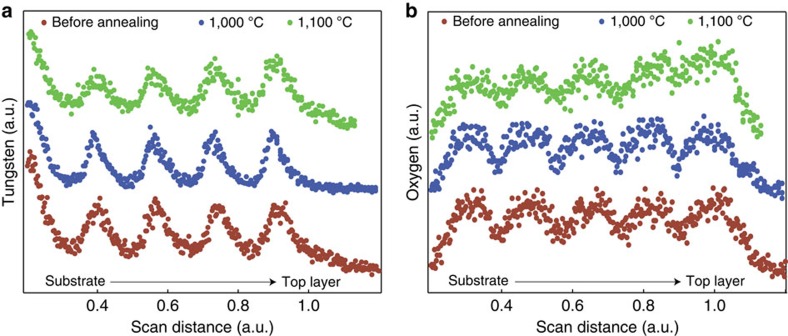
Elemental analysis. EDS linescans of (**a**) tungsten (W-L lines) and (**b**) oxygen (O-K lines) along cross-sections, running from substrate to top layer, of the refractory tungsten–hafnium dioxide metamaterial before annealing (brown), after heat cycling to 1,000 °C (blue), and after heat cycling to 1,100 °C (green). The curves are artificially offset along the *y* axis for clarity. The oxygen figure shows a clear interlayer diffusion as the dips corresponding to tungsten layers are much weaker for the green data set. In contrast, the tungsten figure shows no significant alterations, signalling that the overall multilayer structure is stable beyond 1,100 °C.

As no structural alterations were observed, we conclude that the primary mechanism of degradation in the multilayer structure at 1,100 °C and under vacuum conditions was the diffusion of oxygen from the hafnium dioxide nanolayers and following oxidation of the tungsten nanolayers. This claim is firmly supported by EDS measurements. Figure 6b demonstrates a clear increase in the oxygen concentration in the tungsten nanolayers after heat cycling, while Fig. 6a shows that the tungsten layers are indeed stable. Interestingly, in addition to the noted interlayer diffusion, an overall gradient of oxygen from the vacuum side of the emitter to the tungsten backing is also visible. The presence of this gradient, despite the protective hafnium dioxide capping layer and the use of rough vacuum pressures (2 × 10^−2^ mbar) during the annealing suggests that higher control of vacuum pressures (10^−4^ mbar) or an inert atmosphere are required for long-term thermal stability at temperatures above ∼1,000 °C.

**Figure 7:**
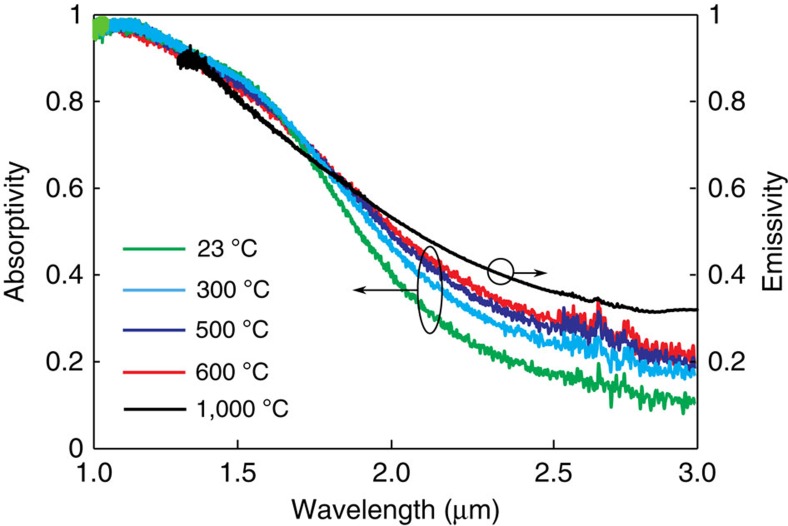
Absorptivity and emissivity at high temperature. Experimental observation of the OTT at high temperatures through normal incidence absorptivity measurements for the tungsten/hafnium dioxide metamaterial emitter at temperatures of 23, 300, 500 and 600 °C. The black line represents the experimental emissivity spectrum at normal incidence of the metamaterial emitter at a temperature of 1,000 °C. The expected slight increase of the electron collision frequency at high temperatures leads to slightly increasing absorptivities and emissivities in metals. The emission peak and the long-wavelength suppression show only a small variation from room temperature up to 1,000 °C, confirming that the topological transition of the metamaterial emitter is thermally robust, and highlighting the potential of the emitter for TPV applications.

Finally, we directly observed the absorption and thermal emission spectrum at high temperatures to verify the spectrally selective excitation of metamaterial modes and presence of the OTT. Kirchhoff's laws dictate that the two spectra have a direct correspondence under critical assumptions of equilibrium. However, temperature dependence of metamaterial parameters is an important issue beyond room temperature. Figure 7 shows the absorptivity spectrum for the tungsten/hafnium dioxide metamaterial at temperatures of 23, 300, 500 and 600 °C as well as a direct measurement of the structure's emissivity at 1,000 °C. The observed spectra confirm the existence of the high-temperature topological transition and show the spectral location of the transition to be thermally robust. The only clear variation in the absorptivity/emissivity measurements as the temperature is increased is a reduction of infrared radiation suppression. This slight increase in measured infrared emission is consistent with the increase of electron collision frequency leading to higher optical losses observed in metals and semiconductors at high temperatures^46^,^47^,^48^,^49^. It is important to note that despite this increase in infrared emissivity the overall roll-off remains evident, indicating that the engineered topological transition of the refractory metamaterial persists until oxidation.

## Discussion

Single pn-junction photovoltaic conversion possesses two primary limitations which restrict the efficiency of solar radiation conversion to the Shockley–Queisser limit^50^. First, free carriers excited by radiation with energy greater than the bandgap thermalize before they can be extracted, creating waste heat. Second, radiation with energy lower than the bandgap is either absorbed as waste heat, or unused, and lost to the casing of the photovoltaic cell, thus deteriorating the overall TPV-efficiency. The motivation of high-efficiency TPV extends from the observation that both of these dominant loss mechanisms are consequences of the spectral width of solar radiation. Specifically, neither restriction occurs at energies slightly greater than the bandgap. To take advantage of high conversion efficiencies in TPV one needs to control the radiative characteristics of the source. The usual solar spectrum is replaced by thermal emission from an engineered selective emitter, suppressing the emission of long-wavelength photons, Fig. 1a. The emission of photons with energies much larger than the bandgap can be avoided by a proper choice of bandgap energy and emitter temperature as the Bose–Einstein occupational factor quickly decays for increasing photon energies.

This concept of TPV employing selective emitters also allows for an expanded range of applications. As the way in which the emitter is heated plays no role in its operation, electrical power can be extracted from any heat source of sufficient temperature. We have calculated the efficiency of a potential TPV system with the presented metamaterial emitter vs. a blackbody emitter. It is assumed that all emitted photons with energies above the bandgap of the PV cell are absorbed and provide electric energy equal to the bandgap energy. The ratio of the generated electric power to the emitted power is the efficiency (called ‘ultimate efficiency'^50^). For an InGaAsSb cell^33^ with a bandgap of 0.55 eV the efficiency at 1,000 °C increases from 19% for a blackbody emitter to 34% for our band-edge metamaterial emitter. Calculations also indicate that the efficiency will further increase above 34% if the emitter temperature can be increased, making clear the need for refractory metamaterials.

In summary, we have provided the experimental evidence of OTTs in the thermal radiation spectrum of an ENZ metamaterial. The wavelength dispersion of material parameters changes the intrinsic thermal energy density and thermal radiation spectrum that we ascertained through high-temperature thermal emission and optical absorption measurements. This spectrum can be of critical use in TPV where the thermal suppression of sub-bandgap photons and enabling of thermal emission above the bandgap boosts the efficiency of energy conversion. Our demonstration of a refractory metamaterial and OTT is in the critical near-infrared range opening the possibility of tuning it to the absorption of a low-bandgap TPV cell. We demonstrated high-temperature stability in the optical/thermal performance till 1,000 °C. Our studies on high-temperature annealing followed by EDS and spectral analysis can pave the way for a systematic approach to analysing high-temperature stability of optical metamaterials. These findings establish a clear path for exploring the unique photonic thermal conductivity and thermal energy density resulting from ENZ effects and hyperboloidal isofrequency surfaces at high temperature.

## Methods

### Metamaterial fabrication

All refractory metamaterial samples were fabricated by magnetron sputtering of the constituent materials onto planar polished silicon substrates under ultrahigh-vacuum conditions. Tungsten layers (20 nm thickness) were deposited by direct current at rate of 0.08 nm s^−1^, while hafnium dioxide layers (100 nm thickness) were deposited by radio frequency sputtering at a rate of 0.21 nm s^−1^.

### Effective medium parameters and ellipsometry measurements

The extracted effective media permittivities values shown were then determined by fitting polarized multi-angle reflection data from a VASE (J.A. Woollam) ellipsometer system. Using WVASE (J.A. Woollam) analysis software, permittivity values for individual layers of tungsten and hafnium dioxide, separate samples, were determined. Using the three layer model (tungsten layer, metamaterial layer and hafnium dioxide capping layer), this data was then assumed for the tungsten substrate and hafnium dioxide capping layer, and the effective medium parameters of metamaterial were obtained from multi-angle reflection data of the complete structure. The extraction technique used for determining optical parameters from the reflection data of the complete emitter is described by Liberman *et al*.^51^.

### Reflection measurements

The specular reflection of the metamaterial between 0.3 and 2.5 μm was measured using a UV/VIS spectrometer (Lambda 1050, Perkin Elmer). For wavelength between 2 and 10 μm a Fourier transform infrared (FTIR) spectrometer (Vertex 70, Bruker) was used to compare the reflection of the sample against a gold mirror reference.

### Annealing of metamaterial samples

In all annealing steps the metamaterial samples were kept under rough vacuum (∼2 × 10^−2^ mbar) in a high-temperature heating stage (TS1500, Linkam) and heated at a rate of 10 °C min^−1^. Samples were maintained at the peak annealing temperature for 3 h before cooling back to room temperature.

### Characterization of absorption from 300 to 600 °C

The absorptivity spectra of the tungsten/hafnium dioxide metamaterial for temperatures from 300 to 600 °C were obtained by collecting reflection measurements using a FTIR microscope (Bruker Hyperion 2000) with 15 × Schwarzschild objective coupled to an FTIR spectrometer (Bruker Vertex 70). The objective operates ∼16.7° off-normal to the surface of the sample and has a collection cone apex angle of ±7°. A gold mirror at room temperature was used as reference.

### Specimen preparation, SEM-imaging and EDS-measurements

The imaging and EDS measurements of the metamaterials were performed on cross-sections prepared by focused ion beam. To enhance the spatial resolution of the EDS-measurements, the accelerating voltage of the SEM was lowered to 5 kV. This energy is sufficient to excite the low energy lines of the present elements (O-K 0.523 keV, Hf-M 1.644 keV, W-M 1.774 keV).

### Characterization of thermal emission at 1,000 °C

Thermal emissivity of the metamaterial at 1,000 °C was obtained using a high-temperature heating stage (TS1500, Linkam) installed at the focus of the FTIR microscope (Bruker, Hyperion 1000) described previously. To calculate the emissivity, it was necessary to divide the thermal emission spectrum by the blackbody spectrum at the same temperature. This step also simultaneously eliminated any artifacts introduced by the setup from the emissivity measurement. As a reference, we replaced the sample with a glassy carbon SIGRADUR G substrate. At elevated temperatures, the glassy carbon is expected to have a wavelength independent emissivity varying between 0.87 and 0.9 in the infrared range.

### Data availability

The authors declare that the data supporting the findings of this study are available within the article.

## Additional information

**How to cite this article:** Dyachenko, P. N. *et al*. Controlling thermal emission with refractory epsilon-near-zero metamaterials via topological transitions. *Nat. Commun.* 7:11809 doi: 10.1038/ncomms11809 (2016).
